# Detection of heat shock protein 27, 70, 90 expressions in primary parenchymatous organs of goats after transport stress by real‐time PCR and ELISA

**DOI:** 10.1002/vms3.327

**Published:** 2020-07-25

**Authors:** Wei Hu, Manxin Fang, Yanzhen Yang, Tian Ye, Ben Liu, Wenya Zheng

**Affiliations:** ^1^ College of Life Science and Resources and Environment Yichun University Yichun China; ^2^ Jiangxi Lvke Agriculture and Animal Husbandry Technology co. LTD Yichun China; ^3^ Engineering Technology Research Center of Jiangxi Universities and Colleges for Selenium Agriculture Yichun University Yichun China

**Keywords:** goat, heat shock proteins, parenchymatous organs, transport stress

## Abstract

Transport stress causes a series of problems to goat production, such as tissue injury and immunity damage. As a pro‐survival pathway, the heat shock response protects healthy cells of goat from stressors. To evaluate the effects of transport stress on heat shock protein (HSPs) expression on goat primary parenchymatous organs, a total of three batches of goats were treated in this study. For each batch, 12 healthy adult male goats were randomly and averagely divided into three groups: Control group (non‐transported group), 2 hr transported group and 6 hr transported group. Real‐time PCR results indicated that the mRNA expression level of heat shock protein 27 (*HSP27*) in all examined organs of 2 hr transport‐treated goats were upregulated (*p* < .05) except lung, and heat shock protein 70 (*HSP70*; except spleen) and heat shock protein 90 (*HSP90*; except liver and lung) were also increased (*p* < .05). In 6 hr transported group, the transcription levels of *HSP27* (except heart and kidney), *HSP70* (except heart, liver and lymph nodes) and *HSP90* (except heart and spleen) were all backed to the original levels or even reduced (*p* < .05). Enzyme‐linked immunosorbent assay (ELISA) results showed that the protein levels of HSP27 (except lymph nodes), HSP70 (except spleen) and HSP90 (except liver and lung) were all increased after 2 hr transport (*p* < .05). After 6 hr transport, HSP27 only in kidney and HSP70 only in heart and liver were upregulated (*p* < .05), while HSP90 in all the examined organs except liver and lung were also maintained in relatively high levels (*p* < .05). Taken together, these results suggested that the expression of HSPs in goat primary parenchymatous organs may be regulated by transport stress time. Moreover, this study also provides some new data to advocate reducing transport stress of goats and improving animal welfare.

## Graphical abstract

This study suggested that the expression of heat shock proteins in goat primary parenchymatous organs may be regulated by transport stress time. Moreover, this study also provides some new data to advocate reducing transport stress of goats and improving animal welfare.

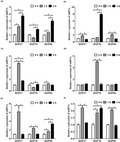

## 1 INTRODUCTION

Transport has become a common method for livestock transfer. Livestock is very susceptible to their environment such as changes in temperature and humidity, hunger and thirst, crowding and so on during transport (He et al., 2019). Stress caused by transport is mainly associated with oxidative stress, which is associated with tissue damage and low immunity (McEwen & Seeman, 1999; Wan et al., 2016; Zou et al., 2016). Transport also can bring certain economic loss to animal husbandry (Hong, Lee, Lee, & Lee, 2019).

Most physiological activities in the body require the participation of parenchymatous organs, which are also very sensitive to different sources of stress (Hudecova et al., 2007; Orenstein & Shamir, 2015; Sasaguri et al., 2017). The level of reactive oxygen species (ROS), as a main inducer of oxidative stress, can be increased by transport stress (Guillonneau et al., 2016; Zou et al., 2016). Many heat shock proteins (HSPs) take part in some stresses, including transport stress (Bao, Sultan, Nowak, & Hartung, 2008; Zhang et al., 2011). HSPs, which are classified into about six families (including the small heat shock proteins (sHSPs, such as HSP27), HSP40, HSP60, HSP70, HSP90 and HSP100 families) based on their monomeric molecular weight, are abundantly expressed in both prokaryotic and eukaryotic organisms (van Eden, van der Zee, & Prakken, 2005). Moreover, previous studies have shown that the expressions of HSP27, HSP70 and HSP90 are significantly changed during transport stress in chickens and pigs, and they are thought to be associated with protective functions (Al‐Aqil & Zulkifli, 2009; Zhang et al., 2011). Therefore, it is reasonable to presume that the expressions of HSP27, HSP70 and HSP90 may change, which play some important protective roles during transport stress in goats. However, there is no documented evidence about the changes in HSP expression in goat parenchymatous organs after transport stress as so far. This information would be of meaningful value to researchers, especially for those who want to minimize the adverse influence of transport stress on animals.

The objective of our study was to assess the expression changes of HSP27, HSP70 and HSP90 in goat parenchymatous organs after transport stress. Based on previous studies, it has been established that expression of HSPs is significantly changed following transport stress, and were determined by real‐time PCR and ELISA assays for mRNA and protein, respectively (Bao et al., 2008). In our study, the expressions of HSP27, HSP70 and HSP90 were upregulated at different levels in order to protect parenchymatous organs from injury after 2 or 6 hr of transport.

## 2 MATERIALS AND METHODS

### 2.1 Animals and treatments

A total of three batches of goats were treated in this study. For each batch, 12 healthy 1‐year‐old West Jiangxi male goats, which were of similar body weight (13.89 ± 2.96 kg), were obtained from western Jiangxi province in this study. These goats were randomly and averagely divided into three groups: a non‐transported group (control group, without transport, *n* = 4), a 2 hr transported group (2 hr group, *n* = 4) and a 6 hr transported group (6 hr group, *n* = 4). Transport treatment was performed as previously described with some modifications (Zhu, Bao, Zhao, & Hartung, 2009). In brief, the transported groups were transported on the road at 28–32°C with the speed of 35–45 km/h and there were no food and water for the goats during transport. No accidents occurred during the transport period. After transportation, goats belonging to all the three groups were anaesthetized with pentobarbital sodium (30 mg/kg) by injection into the jugular vein and subsequently slaughtered in the slaughterhouse. The heart, liver, kidney, lung, spleen and lymph node (mesenteric lymph nodes) samples were collected by sterile surgical instruments. The same organ samples in the different groups of goats were all collected at the same regions. Each organ of every goat was collected from six samples. All the samples were frozen in liquid nitrogen for HSP protein and mRNA expression analyses.

### 2.2 Total RNA Extraction and Real‐time PCR

Real‐time PCR was performed as previously described with modification (Hu et al., 2019). Briefly, the total RNA was isolated from about 20 mg of each tissue sample using the Tissue RNA Kit (R6812, Omega), and reverse‐transcribed into cDNA with the First‐Strand cDNA Synthesis SuperMix Kit (AT301, TransGen). Samples were then stored at −80°C until further analysis. The concentration and purity of the RNA were calculated by a Nanodrop 2000 spectrophotometer (Thermo Fisher). For real‐time PCR, the cDNA was amplified using a Top Green qPCR SuperMix Kit (AQ131, TransGen) on the CFX96 Touch™ Real‐Time System (Bio‐Rad). The following thermal cycling parameters were used: 95°C for 3 min, then 40 cycles of 95°C for 10 s and 60°C for 30 s. Melting curve analysis comprised of the following parameters: 95°C for 10 s, after which the ramp speed was decreased from 1.667°C/s to 0.01667°C/s and data were collected continuously until it reached 95°C where the temperature was held for 30 s, and finally held at 60°C for 15 s. All samples were run in triplicate, whereas non‐template controls were run in duplicate. Primer sequences used were listed in Table 1. The data from real‐time PCR were analysed using the 2^‐ΔΔCt^ method. The relative expression levels were normalized to the expression level of β‐Actin.

**TABLE 1:** Primer sequences used for real‐time PCR

Gene	Sequence (5' to 3')	Product size (bp)	GenBank No.
*HSP27*	F: CCTGGACGTCAACCACTTC R: GCTTGCCAGTGATCTCCAC	76	JQ957566.1
*HSP70*	F: ACGTTCGACGTGTCCATTCT R: TCACCAGCCTGTTGTCGAAG	106	NM_001285703.1
*HSP90*	F: CAAGAGCCTGACCAACGACT R: AAAGGAGCTCGTCTTGGGAC	108	AF548366.1
*β‐Actin*	F: CTCTTCCAGCCTTCCTTCCT R: GGGCAGTGATCTCTTTCTGC	177	NM_001314342.1

Abbreviations: *HSP27*, heat shock protein 27; *HSP70*, heat shock protein 70; *HSP90*, heat shock protein 90.

### 2.3 Protein extraction and enzyme‐linked immunosorbent assay (ELISA)

The total protein was extracted from about 50 mg of each tissue sample using the Total Protein Extraction Kit (P1250, Applygen) according to the manufacturer's instructions. Each tissue contains six samples. In brief, 1 ml of lysis buffer (150 mM NaCl; 50 mM Tris‐HCl, pH 7.5; 1% Triton X‐100; and 0.25% sodium deoxycholate) was added into 50 mg tissue sample and then the pulp refiner was used to get the tissue homogenate. About 500 μl tissue homogenate was transferred to a new 1.5 ml centrifuge tube, and then 1 ml extraction reagent was added into the centrifuge tube. The solution was mixed well and then the upper and lower liquids were discarded after centrifugation. All the tubes were dried at room temperature and dissolved in lysis buffer. The protein concentrations were measured with the Bicinchonininc Acid (BCA) Kit (23225, Thermo Fisher) according to the manufacturer's instructions. Then all the protein samples were adjusted to the same concentration with the protein diluent. The HSP27, HSP70 and HSP90 protein concentrations of tissues were assayed by Goat HSP27, HSP70 and HSP90 ELISA Kits (Ai Pu Rui Sheng) according to the manufacturer's instructions. The detection range of HSP27, HSP70 or HSP90 was 60–1,800 pg/ml, 10–320 pg/ml or 2–90 pg/ml, respectively. Briefly, 50 μl kit standards or 50 μl test samples were added into the well of the enzyme label coated plate and then incubated at 37°C for 30 min. After the well was washed with wash buffer, 50 μl conjugate reagent was incubated at 37°C for 30 min. Then about 50 μl of the colouring reagent A and 50 μl of colouring reagent B were added to the well after washing with wash buffer. The plate was incubated at 37°C for 10 min. Finally, 50 μl of stop solution was added. Optical densities of kit standards and test samples were read at 450 nm using a microplate reader. All samples had six repeats in each experiment. The concentrations of test samples were calculated according to the relation between absorbance values and concentrations of test standards.

### 2.4 Statistical analysis

All data of this study were presented as the mean ± standard deviation (*SD*) and analysed using GraphPad Prism 5.01 software (GraphPad Software Inc.). The SPSS 18.0 software (SPSS Inc.) was used for data analysis, single factor variance analysis was performed after LSD multiple comparisons. The data were statistically analysed by two‐way ANOVA. A *p* value <.05 was considered statistically significant.

## 3 RESULTS

### 3.1 Effects of transport stress on the mRNA expressions of HSP27, HSP70 and HSP90

**FIGURE 1:**
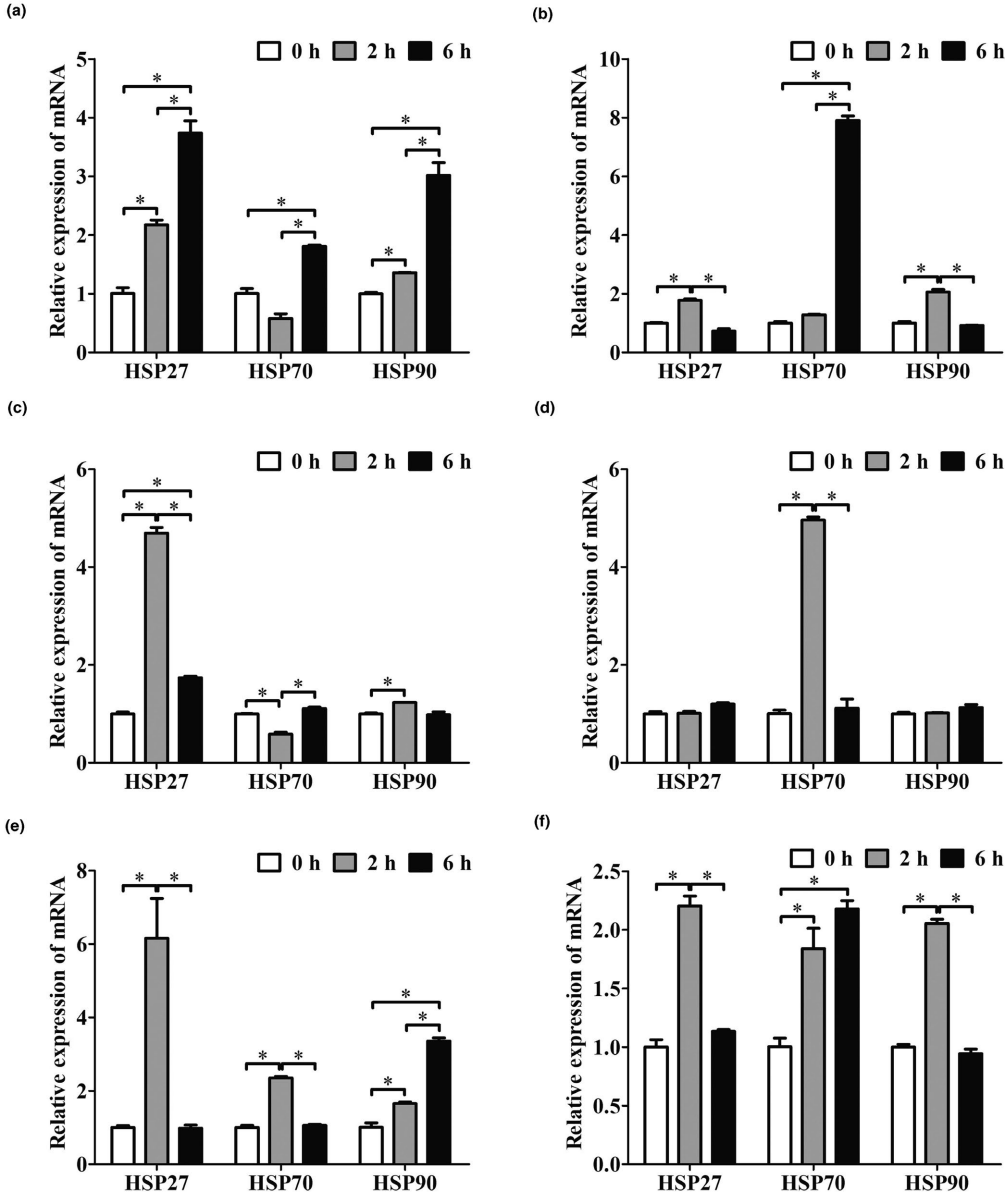
The effects of transport stress on the mRNA expression of heat shock proteins (HSPs) of goat primary parenchymatous organs. Real‐time PCR analysis of the *HSP27*, *HSP70* and *HSP90* mRNA levels in goat heart (a), liver (b), kidney (c), lung (d), spleen (e) and lymph nodes (f) after transport‐treated for 2 and 6 hr. All data are expressed as mean ± *SD* (*n* = 12). *p < .05

Compared to control group, real‐time PCR results showed that the mRNA levels of *HSP27* and *HSP90* were increased in 2 and 6 hr transported groups (*p* < .05), and *HSP70* was only increased in the heart of goats in the 6‐hr group (*p* < .05, Figure 1a). In the liver of goats, the levels of *HSP27* and *HSP90* mRNA were increased in the 2‐hr group (*p* < .05), but decreased after 6 hr transport. On the contrary, the mRNA level of *HSP70* had no change in the 2‐hr group (*p* > .05), but increased after 6 hr transport (*p* < .05, Figure 1b). In the kidney of goats, after 2 hr transport, the mRNA level of *HSP27* and *HSP90* were increased (*p* < .05), while *HSP70* was obviously decreased (*p* < .05). However, *HSP70* was rapidly increased to the control group level after 6 hr transport. Even though the mRNA level of *HSP27* was increased in the 6‐hr group (*p* < .05), the rise was also lower than 2 hr group (*p* < .05). In addition, *HSP70*, *HSP90* was also returned to control group level after 6 hr transport (Figure 1c). In the lung of goats, only *HSP70* mRNA level was increased in the 2‐hr group (*p* < .05), but immediately decreased after 6 hr transport. Both *HSP27* and *HSP90* were not changed in two transport groups (*p* > .05, Figure 1d). In the spleen of goats, the mRNA levels of *HSP27* and *HSP70* were highly increased in the 2‐hr group (*p* < .05), but decreased after 6 hr transport and *HSP90* was upregulated by transport from 2 to 6 hr (*p* < .05, Figure 1e). In the lymph nodes of goats, the expression of *HSP27*, *HSP70* and *HSP90* mRNA were obviously increased after 2 hr transport (*p* < .05), but *HSP27* and *HSP9*0 were decreased after 6 hr transport. The expression of *HSP70* was continually upregulated by transport treatment for 6 hr (*p* < .05, Figure 1f). These results suggested that *HSP27* and *HSP90* were mainly involved in the regulation of short‐term transport except lung, but *HSP70* was a special case which was involved in both short‐ (lung, spleen and lymph nodes) and long‐term (heart, liver and lymph nodes) transport.

### 3.2 Effects of transport stress on the protein expressions of HSP27, HSP70 and HSP90

**FIGURE 2:**
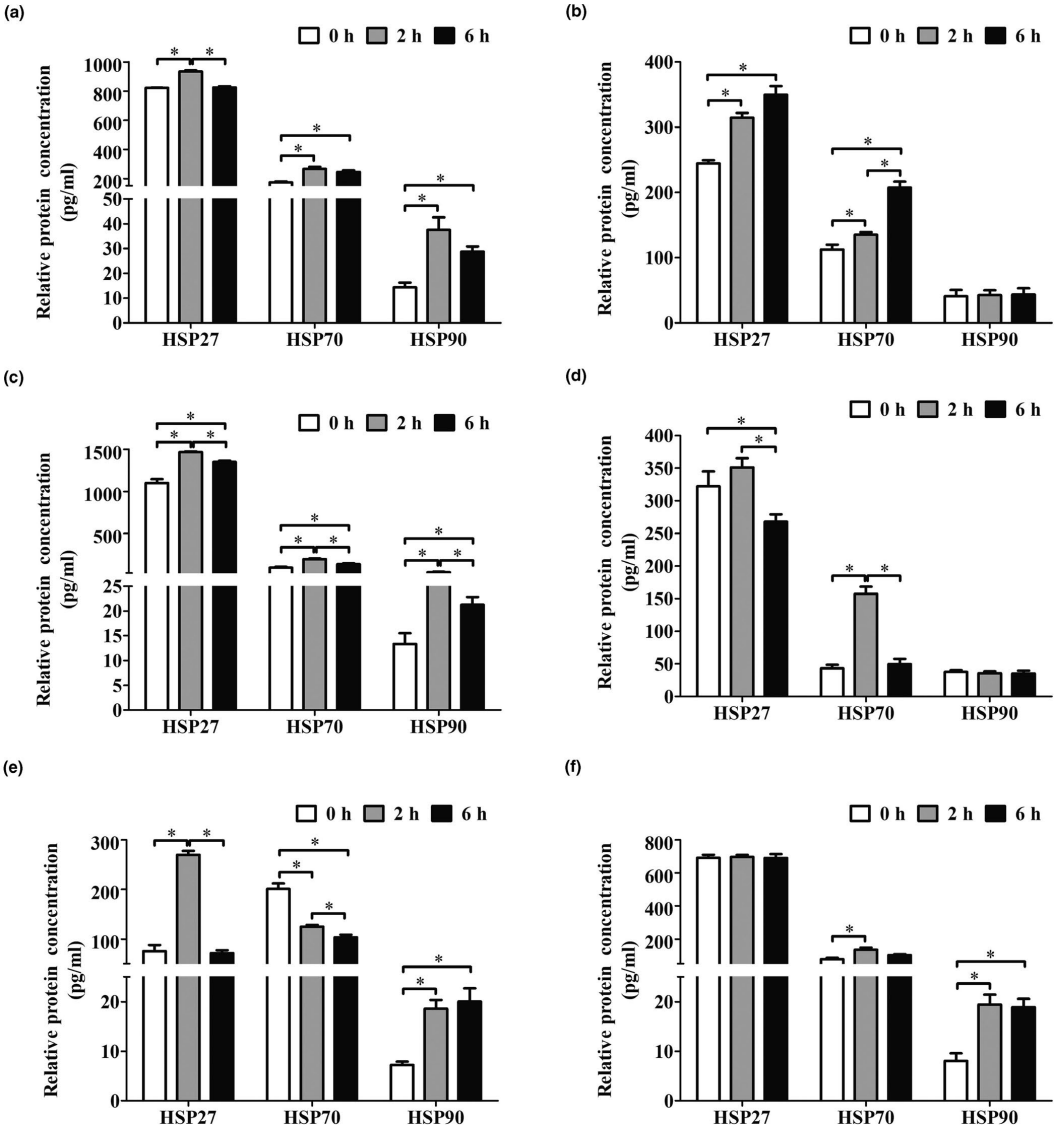
The effects of transport stress on heat shock proteins (HSPs) protein expression of goat primary parenchymatous organs. ELISA analysis of the HSP27, HSP70 and HSP90 protein levels in goat heart (a), liver (b), kidney (c), lung (d), spleen (e) and lymph nodes (f) after transport‐treated for 2 and 6 hr. All data are expressed as mean ± *SD* (*n* = 12). *p < .05

Next we analysed the protein expressions of HSP27, HSP70 and HSP90 in goat primary parenchymatous organs after transport stress by using ELISA. In the heart of goats, the protein expressions of HSP27, HSP70 and HSP90 were increased in the 2 hr group when compared to the control group (*p* < .05). Then the expressions of HSP27 and HSP90 proteins were decreased after 6 hr transport when compared to the 2 hr group (*p* < .05), but HSP70 was maintained the same level between 6 and 2 hr group. Otherwise, the protein level of HSP90 in the 6‐hr group was higher than control group (*p* < .05), and HSP27 was equal to the control group (*p* > .05, Figure 2a). In the liver of goats, the expressions of HSP27 and HSP70 proteins were upregulated after 2 or 6 hr transport (*p* < .05), but not HSP90 (*p* > .05, Figure 2b). In the kidney of goats, the protein expressions of HSP27, HSP70 and HSP90 were increased in the 2‐ and 6‐hr groups and the levels of these proteins in the 6‐hr group was lower than the 2‐hr group (*p* < .05, Figure 2c). In the lung of goats, only HSP70 was increased after 2 hr transport (*p* < .05), but quickly decreased after 6 hr transport when compared to the 2 hr group (*p* < .05). Otherwise, HSP27 was significantly decreased after 6 hr transport (*p* < .05). And HSP90 had no changes after transport (*p* > .05, Figure 2d). In the spleen of goats, HSP27 and HSP90 were increased in the 2‐hr group (*p* < .05). In the 6‐hr group, HSP27 was decreased to the control group level. However, the expression of HSP70 protein was downregulated after 2 or 6 hr transport (*p* < .05, Figure 2e). In the lymph nodes of goats, the protein levels of HSP70 and HSP90 had an increase in the 2‐hr group (*p* < .05). After 6 hr transport, HSP70 was decreased to control group level but HSP90 was higher than the 2 hr group (*p* < .05). In Bbth the 2 and 6 hr groups, HSP27 had no changes (*p* > .05, Figure 2f). These results suggested that HSP27 was mainly involved in the regulation of short‐term transport, which was consistent with the mRNA results. As for HSP90, it was involved in both short‐ and long‐term transport except liver and lung, but the protein level was decreased in heart and kidney during long‐term transport when compared to the short‐term. The HSP70 protein was subjected to similar trends with the mRNA changes after transport stress.

## 4 DISCUSSION

In this study, we used two kinds of experimental methods to test the expressions of HSP27, HSP70 and HSP90 in the goat primary parenchymatous organs after transport stress. The relative quantification of mRNA encoding for three different HSPs and protein level were measured by real‐time PCR and ELISA, respectively. Our results showed that the changes of HSP27, HSP70 and HSP90 expressions in goat primary parenchymatous organs can be caused by transport stress. The differences of HSP expression patterns among primary parenchymatous organs after transport stress may be related to the sensitivity of different organs to transport stress.

In stressed animals, the myocardial contractility disorders are frequently occurring than normal animals (Perfilova, Sadikova, Prokof'ev, Inozemtsev, & Tyurenkov, 2016). Moreover, some arrhythmic diseases of the heart are often induced by stress (Meerson, 1994). Thus, stress has become a major cause of heart disease so far. Previous studies have demonstrated that heat shock proteins (HSPs) involved in some stress processes such as the increase of HSP60, HSP70 and HSP90 mRNA and protein levels were observed in the heart of broiler (Yu, Bao, Yan, & Lei, 2008). The mRNA and protein levels of HSP70 were increased in the heart of pig after transport stress (Yu, Bao, Zhao, & Lv, 2007). Meanwhile, it also has been reported that over‐expression of HSP27 could provide significant resistance to reduce heat shock and oxidative stress (Hao, Zhang, Timakov, & Zhang, 2007). In our study, we also found that the mRNA and protein expressions of HSP27, HSP70 and HSP90 were increased in the heart of transport‐treated goats. Therefore, our data implied that heart may be one of the most invulnerable organs during transport stress.

The liver and kidney, as the main organs of animal, have some functions that cannot be compensated by other organs. Many types of stresses such as endoplasmic reticulum stress and oxidative stress can cause some damages to the liver and kidney (Gallazzini & Pallet, 2018; Geng et al., 2013; Yang et al., 2016; Yao et al., 2015). A previous study has demonstrated that HSP70 protein was increased in the kidney of pig after transport stress (Yu et al., 2007). The HSP/chaperone network is a major component of multiple stress responses (Jacob, Hirt, & Bendahmane, 2017). Another research has demonstrated that heat stress can cause both liver and kidney injury, which accompanied with increase expression of HSP70 (Roncal‐Jimenez et al., 2018). In the present study, we observed the upregulation of HSP27 and HSP90 mRNA and protein levels in goat liver or kidney after 2 hr transport, but the mRNA or protein levels of these two molecules were sharply decreased after longer time transport. Accordingly, it is reasonable to postulate that HSP27 and HSP90 play some important regulatory roles during short‐term transport stress, which is worthy of further investigation. In the liver, HSP70 level was not significantly increased during short‐term transport stress, but it was obviously increased after longer time transport. In kidney, the expression of HSP70 only had slight changes. Therefore, these results suggested that HSP27 and HSP90 may play positive roles during short‐term transport stress, and HSP70 mainly participated in long‐term transport stress. Taken together, we realized that kidney may easily be injured than liver during longer time transport.

Stress can cause emphysema, lung cellular destruction and lung epithelial cells apoptosis in humans (Ahamed, Akhtar, Khan, Alhadlaq, & Aldalbahi, 2017; Tuder et al., 2003). The heat shock response that induces HSP expression is an endogenous mechanism to protect cells from injury. The increase of HSP70 expression can be found during lung injury (Lyons, Raj, Chittams, Kilpatrick, & Deutschman, 2015). In our study, we also found that mRNA and protein levels of HSP70 in goat lung were increased after 2 hr transport. Otherwise, protein expression of HSP27 was increased during heat stress in human lung, but no significant increase in goat lung was observed after transport stress (Doberentz, Genneper, Wagner, & Madea, 2017). As for lung, only HSP70 was increased in the short‐term transport, but decreased to the initiating level after longer time transport. Therefore, we may conclude that the lungs are the most vulnerable organs subjected to transport stress damage.

Spleen and lymph nodes are the main immune organs of the animal. The immune tolerance of spleen can be influenced by stress (Sasaguri et al., 2017). The weights of spleen and bursa of Fabricius are increased in laying hens due to transport stress (Matur et al., 2016). When chicken suffered heat stress, the expression of HSP27 and HSP70 were obviously increased in chicken spleen (Xu, Li, Huang, He, & Tian, 2014). In our study, the mRNA expressions of *HSP27*, *HSP70* and *HSP90* in goat spleen were markedly increased after being transport‐treated for 2 hr, and *HSP90* was increased more after 6 hr transport. Protein expressions of HSP27 and HSP90 in goat spleen were also increased after transport stress, but HSP70 was significantly decreased. It is may be due to the degree of goat spleen damage exceeding the regulatory protection ability of HSP70. These data suggested that HSP27 and HSP90 played critical roles in spleen during short‐term transport stress. However, HSP90 was responsible for regulating the stress of longer time transport. Moreover, we further showed that mRNA expressions of *HSP27*, *HSP70* and *HSP90* in goat lymph nodes were significantly increased after short‐term transport stress. However, only HSP70 and HSP90 proteins were increased, which might be due to the inhibition of protein translation of HSP27 during this process. Our data suggested that HSP70 and HSP90 may play some key roles during short‐term transport stress in lymph nodes of goats. For longer time transport, the expression of HSP70 and HSP90 were not synchronized between mRNA and protein level. However, there was no article that had illustrated so far. Therefore, the effect of long‐term transport stress on lymph nodes of goats need to be further investigated.

The compromised intracellular environment can induce cell apoptosis at last under stressful situations. Many studies have found that HSP27, HSP70 and HSP90 signalling pathways are involved in the regulation of apoptosis in order to promote cell survival (Fernández, Ordóñez, Reiter, González‐Gallego, & Mauriz, 2015; Gao et al., 2015; Xu et al., 2017; Zheng et al., 2018). The decrease of HSPs levels indicated that the transport stress may lead to excessive damage making different kind of cells lose the repair mechanisms. Therefore, whether HSP27, HSP70 and HSP90 are involved in regulating apoptosis to protect organs from damage that caused by transport stress is worthy of further investigation.

## 5 CONCLUSION

In summary, our data indicated that HSP27 and HSP90 are preferentially increased during early short time transport and HSP70 is increased after longer time transport in most primary parenchymatous organs of goats, but the specific regulatory signalling pathways still need to be further explored. Perhaps the lung is the most vulnerable organ and the heart may be the toughest one during transport stress. Further research will focus on the exploration of specific protective mechanism. Otherwise, this study also can provide new data for the research of improvement of animal welfare.

## ANIMAL WELFARE STATEMENT

The animal care and experimental procedures used in this study conformed to the regulations and guidelines of the regional Animal Ethics Committee and the Ethical and Animal Welfare Committee of Yichun University.

## CONFLICT OF INTEREST

I certify that neither I nor my co‐authors have a conflict of interest as described above that is relevant to the subject matter or materials included in this work.

## AUTHOR CONTRIBUTION

**Wei Hu:** Conceptualization; Data curation; Formal analysis; Investigation; Methodology; Software; Writing‐original draft. **Manxin Fang:** Data curation; Investigation; Methodology; Software. **Yanzhen Yang:** Data curation; Investigation; Methodology; Software. **Tian Ye:** Data curation; Formal analysis; Methodology; Software. **Ben Liu:** Conceptualization; Data curation; Funding acquisition; Project administration; Resources; Supervision; Visualization; Writing‐original draft; Writing‐review & editing. **Wenya Zheng:** Conceptualization; Data curation; Investigation; Project administration; Supervision; Writing‐original draft; Writing‐review & editing.

### Peer Review

The peer review history for this article is available at https://publons.com/publon/10.1002/vms3.327.
